# Transcriptome Analysis Reveals Candidate Genes and Regulatory Pathways Involved in Pepper Seedlings (*Capsicum annuum* L.) Response to Short-Term High-Temperature Stress

**DOI:** 10.3390/biology15211925

**Published:** 2026-10-23

**Authors:** Wen Wang, Xinyue Cui, Huilin Chen, Xiangxiang Zhang, Junqiang Wang, Nengbing Hu, Meirong Ye, Shoujing Zhu

**Affiliations:** College of Agriculture, Anhui Science and Technology University, Chuzhou 233100, China

**Keywords:** pepper, high-temperature stress, transcriptome analysis, heat-tolerant pepper lines, heat-sensitive pepper lines

## Abstract

Global climate change and warming are increasing challenges to plant growth, development, and crop productivity. To investigate the responses of pepper seedlings to short-term high-temperature stress, we performed time-course physiological and transcriptome analyses on leaves of a heat-tolerant line (J18-1) and a heat-sensitive line (J190) at the 6–8 true leaves stage after exposure to 42 °C for 0, 6, and 24 h. Comparisons were made within each line using 0 h as the baseline. Under high-temperature stress, J18-1 maintained chlorophyll without degradation, with SOD, POD, and CAT activities increasing at 6 h and remaining elevated at 24 h, and MDA content remaining low. In contrast, J190 showed a transient chlorophyll increase that returned to the baseline at 24 h, with antioxidant enzyme activities increasing at 6 h but declining at 24 h, and MDA content increasing at 6 h and remaining elevated at 24 h. In total, 16,912 differentially expressed genes (DEGs) were identified across all comparisons. GO and KEGG enrichment analyses indicated that protein processing in the endoplasmic reticulum and plant hormone signal transduction were the most significantly enriched pathways, in which *HSP20* and *ERF* genes showed genotype-dependent expression changes. qRT-PCR confirmed that *HSP20* genes were up-regulated at both 6 h and 24 h under high-temperature stress, consistent with the transcriptome data. These results provide candidate genes and pathways for elucidating pepper seedling responses to short-term high-temperature stress.

## Simple Summary

High-temperature stress limits the yield and quality of pepper (*Capsicum annuum* L.), especially under protected cultivation. We performed physiological and transcriptome analyses on two pepper lines with divergent heat tolerance. The tolerant line kept its chlorophyll and antioxidant defenses active with little membrane damage, whereas the sensitive line responded briefly and suffered increasing damage. GO and KEGG enrichment analyses uncovered conserved core heat-responsive pathways exhibiting genotype-dependent transcriptional dynamics. Small heat-shock protein (*HSP20*) genes were strongly induced and confirmed by qRT-PCR. This study characterizes pepper seedlings’ transcriptional responses to heat and provides candidate genes for breeding heat-tolerant peppers.

## 1. Introduction

Pepper (*Capsicum annuum* L.), a vegetable crop with a long cultivation history, is widely cultivated worldwide and highly favored as a culinary ingredient in diverse global cuisines [1]. Beyond its dietary uses, pepper has a broad spectrum of applications. It serves not only as a source of natural dyes and a component in cosmetics but also as a vital component in pharmaceutical preparations and an effective ingredient in insect repellents [2]. Furthermore, pepper contains abundant nutrients, minerals, and vitamins. However, while pepper thrives in warm climates, it is highly susceptible to high-temperature stress. When ambient temperatures exceed 35 °C, pepper plants experience various developmental challenges, including delayed floral bud differentiation, abnormal development of floral organs, reduced pollen viability, and impaired fertilization [3,4]. Prolonged high temperatures can also induce cellular damage, such as protein misfolding and aggregation, membrane injury, disruption of microtubule organization, and accumulation of reactive oxygen species [5]. Ultimately, these adverse effects hinder plant growth and development, significantly compromising both pepper yield and quality.

In recent years, the rapid expansion of protected agriculture has led to a continuous increase in the cultivation area of pepper plants grown under controlled environments. When temperatures inside these controlled environments exceed 35 °C, pepper plants are subjected to high-temperature stress [6,7]. Therefore, identifying key genes involved in the high-temperature stress response of pepper holds significant implications for enhancing heat tolerance and breeding heat-resistant pepper varieties. China has the largest pepper cultivation area in the world, and pepper generates the highest annual economic value among all vegetables [8]. As global temperatures continue to rise, high-temperature stress has become a critical agricultural challenge worldwide [9]. Future extreme climate events may have severe impacts on crop growth and yield. As pepper is one of the most important horticultural crops, developing new varieties with enhanced stress tolerance and adaptability will facilitate more effective responses to climate change. By elucidating the relationship between heat-tolerance phenotypes and genetic regulation in pepper, we can uncover the underlying protective mechanisms and adaptive pathways under high-temperature stress, thereby providing theoretical support and technical guidance for breeding new varieties.

Transcriptomics enables the precise elucidation of dynamic changes in gene expression and gene regulatory networks, providing critical clues for identifying key heat-tolerant genes. Through transcriptome analysis of pepper under high-temperature stress, heat-responsive genes can be reliably identified. Metabolomics, which characterizes changes in metabolites and metabolic pathways, provides in-depth insights into heat-tolerance-related metabolic alterations by profiling metabolite abundance in pepper under high-temperature stress, thereby laying a foundation for deciphering the molecular mechanisms of heat-tolerance in pepper. In recent years, transcriptome sequencing combined with bioinformatics analysis has led to the identification of multiple gene families associated with the high-temperature stress response in pepper, such as the *HSF*, *HSP*, *WRKY*, and *NAC* gene families [10,11,12].

In recent studies, the heat-tolerant pepper variety 17CL30 and the heat-sensitive variety 05S180 were selected as experimental materials to analyze transcriptomic changes in the leaves of pepper seedlings under 40 °C high-temperature stress. The results showed that under high-temperature stress, 5754 and 5756 DEGs were identified in the heat-tolerant line 17CL30 and the heat-sensitive line 05S180, respectively. Integrated metabolomic analysis revealed that genes of the *GST* (glutathione S-transferase) family play a crucial role in this process [13]. Transcriptomic analysis of pepper seedlings treated at 40 °C indicated that 29,249 DEGs could be clustered into 12 groups with distinct expression dynamics. Among 25 *HSFs*, five showed significant responses to high-temperature stress. Subsequently, transcriptomic analysis was performed under high-temperature stress using the heat-sensitive pepper variety S590 (CaS) and the heat-tolerant variety R597 (CaR). A total of 3799 and 4010 DEGs were identified in R597 (CaR) and S590 (CaS), respectively. Among these, several DEGs associated with transcription factors and plant hormone signaling pathways showed notably higher expression levels in R597 than in S590 [14]. These findings provide a solid foundation for further exploration of the molecular mechanisms underlying high-temperature stress response across different pepper genotypes.

Thus, in this study, the “J-series” pepper lines developed by the Pepper Research Group at Anhui Science and Technology University were used as experimental materials. After preliminary screening at 42 °C for 48 h, heat-tolerant and heat-sensitive lines were identified. Physiological and biochemical indicators were measured in leaves of both heat-tolerant and heat-sensitive lines at the 6–8 true leaves stage under high-temperature stress. Time-series transcriptome analyses were performed at 0 h, 6 h, and 24 h, enabling the identification of time-dependent DEGs. Gene Ontology (GO) and Kyoto Encyclopedia of Genes and Genomes (KEGG) pathway enrichment analyses of these DEGs suggested that protein processing in the endoplasmic reticulum and plant hormone signal transduction may be two important pathways involved in the response of pepper to high-temperature stress. The potential mechanisms underlying the responses of these pathways to high-temperature stress were then analyzed. In addition, *ERF* family genes and *HSP20* family genes of pepper were suggested to play roles in the response to high-temperature stress. These findings may provide a foundation for elucidating the physiological, biochemical, and molecular mechanisms of pepper under high-temperature stress.

## 2. Materials and Methods

### 2.1. Plant Materials and Growth Conditions

Twenty-one “J-series” pepper accessions were collected from the pepper germplasm resource repository of Anhui Science and Technology University. Seed vigor was assessed to ensure the uniformity of the experimental materials used in subsequent experiments. Plants were grown in an artificial climate chamber under a 16 h light/8 h dark photoperiod, with a photosynthetic photon flux density (PPFD) of 200 μmol·m^−2^·s^−1^. The growth temperature was maintained at 25 °C, and the relative humidity was maintained at 70%.

The selected seeds were sown in 32-cell plug trays. When the seedlings reached the two-true-leaf and one-heart stage, they were transplanted into nursery pots of uniform size. When the plants reached 6–8 true leaves, plants with uniform growth were selected for high-temperature treatment. The artificial climate chamber was thoroughly inspected, and its temperature control system was calibrated to accurately maintain a high-temperature environment at 42 ± 0.5 °C. All other conditions remained unchanged. The selected plants were transferred to another artificial climate chamber and first acclimated for one day at 30 °C with other conditions unchanged; the temperature was then set to 42 °C and maintained for 48 h. During the treatment, plant status was observed at regular intervals, and phenotypic changes such as leaf wilting and curling were recorded. After the treatment, heat tolerance was comprehensively evaluated based on multiple indicators. This screening experiment was performed in duplicate. The results of the two heat injury indices are shown in the Supplementary Materials.

(1) $$\mathrm{HI}=\frac{\sum (\mathrm{xi}\times \mathrm{ai})}{4N}\times 100\%$$

Heat Injury Index Calculation:

HI—heat injury index;

xi—number of plants at each injury grade;

ai—value of each injury grade;

N—total number of investigated plants.

The heat injury was divided into five grades according to the following criteria:

Grade 0: plants grow normally with no injury symptoms; Grade 1: slight wilting of plants, with mild dehydration observed in a few young leaves; Grade 2: plants show wilting, with drooping and wrinkling of middle and lower leaves; Grade 3: severe wilting of plants, with pronounced drooping of upper leaves, dehydration, and wrinkling of young leaves; Grade 4: all leaves of the plant are dried or shed.

### 2.2. Physiological Index Determination

At the 6–8 true leaves stage, heat-tolerant and heat-sensitive pepper lines were subjected to high-temperature treatment for 0, 6, and 24 h. For physiological measurements, each biological replicate consisted of a pooled leaf sample from multiple independently cultivated plants. Specifically, for each treatment condition, plants of uniform growth were selected. For each biological replicate, multiple plants were randomly selected, and equal amounts of leaf tissue were collected and pooled into one sample. The plants used for the three biological replicates were independent. Before acclimation and high-temperature treatment, plants were randomly assigned to the treatment groups.

Functional leaves (the 3rd–4th leaves from the top) were collected and immediately frozen in liquid nitrogen, with three biological replicates for each sample. Leaf chlorophyll content was determined by 95% ethanol extraction followed by ultraviolet spectrophotometry. The contents of chlorophyll a, chlorophyll b, and total chlorophyll were calculated based on absorbance values at characteristic wavelengths. Antioxidant enzyme activities, including superoxide dismutase (SOD), peroxidase (POD), and catalase (CAT), and malondialdehyde (MDA) content were quantified using commercial assay kits (Solarbio, Beijing, China). Fresh leaves were ground on ice to prepare crude enzyme extracts, and all procedures were conducted strictly according to the manufacturer’s instructions. Absorbance values were measured by spectrophotometry, and the enzyme activities and MDA content were calculated.

### 2.3. RNA-Sequencing

Total RNA was extracted from leaf samples of the control and high-temperature-treated plants using a Polysaccharide & Polyphenol Plant Total RNA Kit (Borez, Nanjing, China) according to the manufacturer’s instructions. RNA concentration and quality were assessed using a NanoDrop 2000 spectrophotometer (Thermo Fisher Scientific, Waltham, MA, USA) and Agilent 2100 Bioanalyzer with an RNA 6000 Nano Kit (Agilent Technologies, Santa Clara, CA, USA). mRNA was enriched using PrimeScript™ RT reagent Kit with gDNA Eraser (Perfect Real Time) (Takara, Beijing, China) and fragmented using divalent cations at high temperature. After repair, A-tailing, and adapter ligation, the cDNA library was amplified and purified. For all physiological measurements and RNA-seq, three biological replicates were used. For RNA-seq, each biological replicate consisted of leaves from a single independently cultivated plant, with samples collected and stored separately for each plant.

Library quality was initially assessed using Qubit 2.0, and insert sizes were verified using the Agilent 2100 Bioanalyzer. Quantitative PCR (qPCR) was then used to measure effective library concentration. High-throughput sequencing was performed using paired-end 150 bp reads on the Illumina HiSeq™ 2500 platform (Illumina, Inc., San Diego, CA, USA). All sequencing data have been submitted to the NCBI Sequence Read Archive (SRA) database under BioProject accession number PRJNA1290848.

### 2.4. Differential Gene Expression Analysis

Raw sequencing reads were filtered to remove low-quality reads (defined as those with >20% of bases having quality scores < 10), adapter sequences, and reads containing more than 5% ambiguous bases (N). Raw sequencing data were subjected to quality-control processing using FastQC (version 0.11.5). Trimmomatic was used for data filtering to remove adapter sequences from the read ends, and reads shorter than 20 bp were discarded. The filtered data were then subjected to quality-control assessment using FastQC (version 0.11.5). *Capsicum annuum* reference genome (cultivar CM334, genome version Pepper_CM334_v1.55) using HISAT2 (version 2.2.1) with default parameters. Uniquely mapped reads were filtered and deduplicated for downstream analysis.

Functional annotation of assembled transcripts was performed using BLASTX (BLAST+ 2.15.0+) (E-value threshold = 1 × 10^−5^) against multiple databases: NCBI Nr, Nt, Swiss-Prot, PFAM, Gene Ontology (GO), EuKaryotic Orthologous Groups (KOG), and Kyoto Encyclopedia of Genes and Genomes (KEGG). DEGs were identified using DESeq2 (v1.34.0). Genes with a false discovery rate (FDR)-adjusted *p*-value < 0.05 and |log_2_ fold change| ≥ 2 were considered significant DEGs. GO and KEGG enrichment analyses were conducted using topGO (v2.50.0) and KOBAS (v3.0), respectively, with significance defined as adjusted *p* ≤ 0.05. Visualization of DEGs and enriched pathways was performed using the pheatmap R package (v1.0.12) in RStudio (v2025.05.0).

### 2.5. qRT-PCR Analysis

Quantitative real-time PCR (qRT-PCR) was performed using TB Green^®^ Premix Ex Taq™ II (Takara, Beijing, China) according to the manufacturer’s protocol on a ViiA 7 Real-Time PCR System (Thermo Fisher Scientific, Shanghai, China). Each reaction contained 1 μL of cDNA (derived from CK or high-temperature treatments), 1 μL each of forward and reverse primers (10 μM), 10 μL of TB Green Fast qPCR Mix, and 7 μL of RNase-free water. Primers were designed using Primer 5.0 software and are listed in Supplementary Materials. CaActin served as the internal reference gene. Relative mRNA expression levels were calculated using the 2^−∆∆Ct^ method.

A total of three biological replicates were used for qRT-PCR. Each biological replicate consisted of a pooled sample of three independent plant samples of RNA-seq, with RNA extracted separately for each biological replicate. Three qRT-PCR technical replicates were performed for each pooled RNA sample. The RNA samples used for qRT-PCR were the same batch of pooled RNA samples used for RNA-seq, rather than those obtained from another independent stress experiment.

Data processing: For each biological replicate, the Ct values of the three technical replicates were first averaged and used as the gene expression level of that biological replicate; subsequent statistical analyses and standard deviation calculations were based on the means of the three biological replicates.

### 2.6. Statistical Analysis

One-way analysis of variance (ANOVA) followed by Tukey’s multiple comparisons test was performed using GraphPad Prism 9.5. *p* < 0.05 was considered statistically significant. Asterisks (*) denote significant changes compared to 0 h (*p* < 0.05). Double asterisks (**) denote significant changes compared to CK (*p* < 0.01). Data are means of three replicates.

## 3. Results

### 3.1. High-Temperature Stress in Pepper

**Figure 1:**
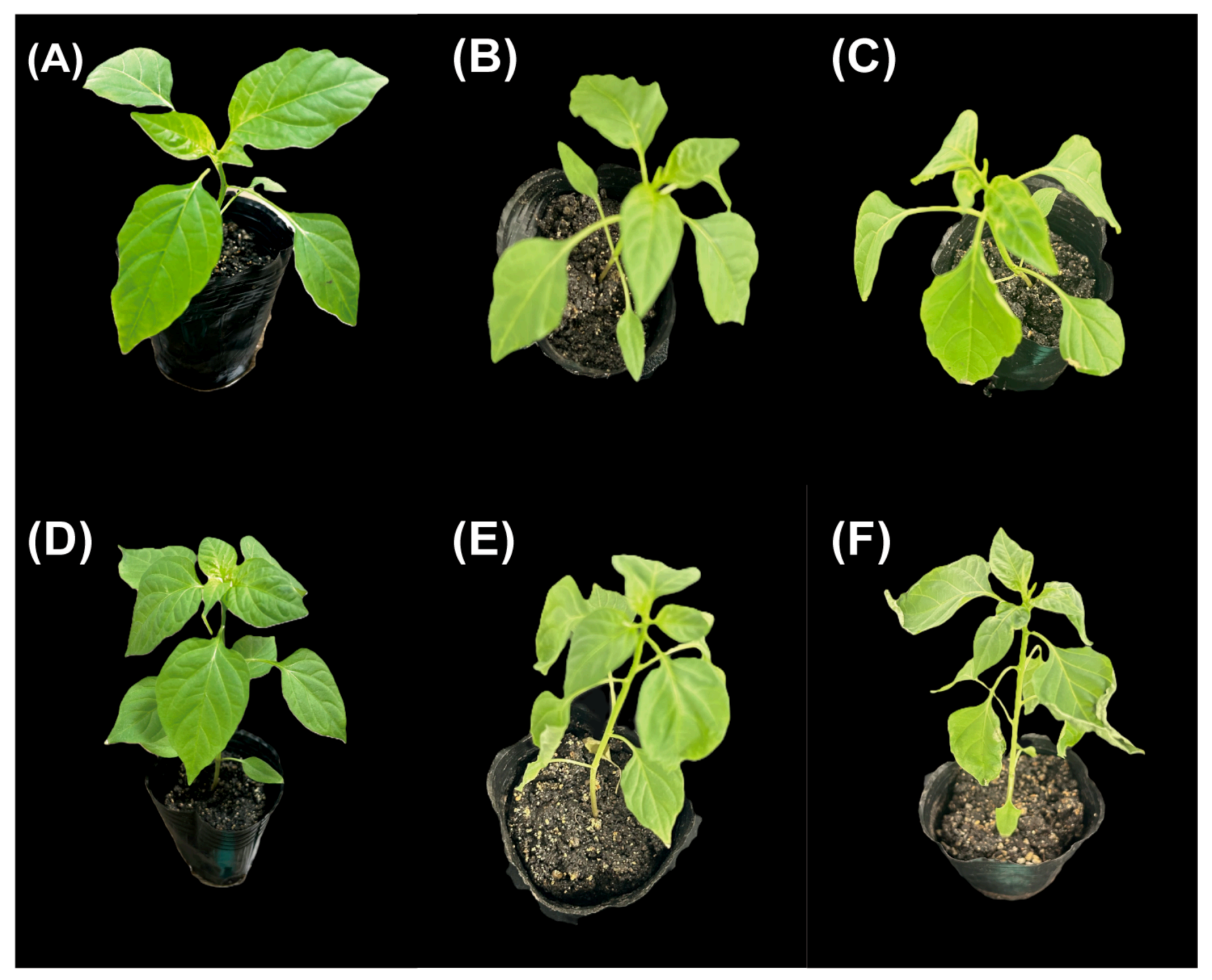
Phenotypic characteristics of the heat-tolerant pepper line J18-1 and the heat-sensitive line J190 under 42 °C high-temperature stress for different durations. (**A**) Heat-tolerant pepper line J18-1 under 42 °C for 0 h. (**B**) Heat-tolerant pepper line J18-1 under 42 °C for 6 h. (**C**) Heat-tolerant pepper line J18-1 under 42 °C for 24 h. (**D**) Heat-sensitive line J190 under 42 °C for 0 h. (**E**) Heat-sensitive line J190 under 42 °C for 6 h. (**F**) Heat-sensitive line J190 under 42 °C for 24 h.

Twenty-one “J-series” pepper germplasm accessions, collected from the Pepper Germplasm Resource Bank of Anhui Science and Technology University, were subjected to high-temperature treatment at 42 °C for 48 h. During treatment, plant status was monitored periodically, and phenotypic changes such as leaf wilting and curling were recorded. After treatment, heat tolerance was comprehensively assessed. Based on the heat-tolerance index formula, J18-1 was identified as a heat-tolerant line and J190 as a heat-sensitive line under 42 °C treatment for 48 h (Table 1) (Figure 1).

### 3.2. Measurements of Chlorophyll Content, Antioxidant Enzyme Activities, and MDA Content

**Figure 2:**
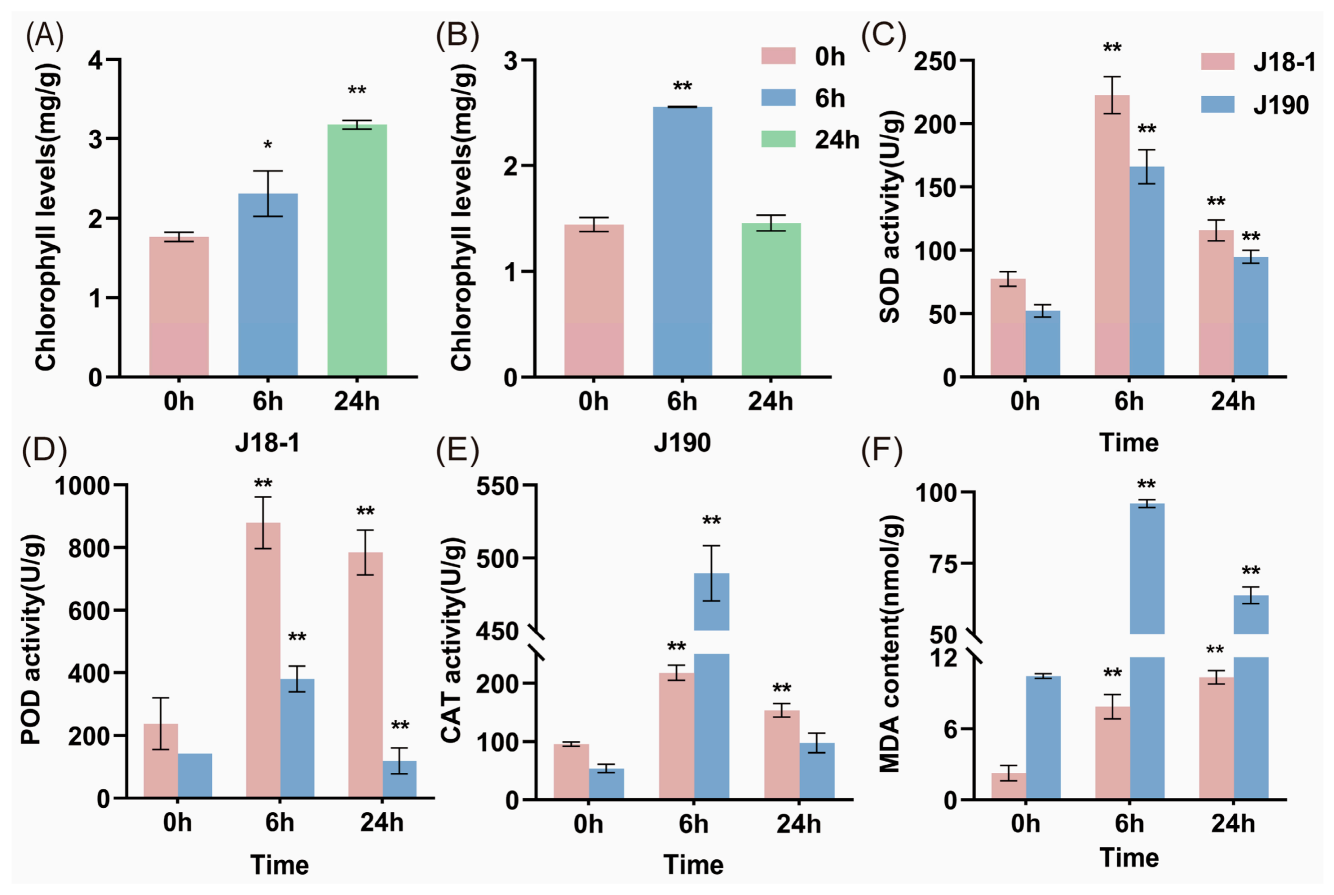
Determination of Physiological Indices in Heat-Tolerant Line J18-1 and Heat-Sensitive Line J190 under 42 °C High-Temperature Stress for Different Durations. (**A**,**B**) Changes in chlorophyll content in the heat-tolerant pepper line J18-1 and heat-sensitive pepper line J190 at 42 °C for different durations. (**C**) Changes in SOD activity in the heat-tolerant line J18-1 and the heat-sensitive line J190 under 42 °C for different durations. (**D**) Changes in POD activity in the heat-tolerant line J18-1 and the heat-sensitive line J190 under 42 °C for different durations. (**E**) Changes in CAT activity in the heat-tolerant line J18-1 and the heat-sensitive line J190 under 42 °C for different durations. (**F**) Changes in MDA content in the heat-tolerant line J18-1 and the heat-sensitive line J190 under 42 °C for different durations. Asterisks (*) denote significant changes compared to CK (*p* < 0.05). Double asterisks (**) denote significant changes compared to CK (*p* < 0.01). Data are means of three replicates.

We measured chlorophyll content at 6 h and 24 h under high-temperature stress (Figure 2A,B). In the heat-tolerant line J18-1 (Figure 2A), total chlorophyll content was higher than that of the control (0 h), showing a stable and consistently increasing trend. In the heat-sensitive line J190 (Figure 2B), chlorophyll content increased significantly at 6 h under 42 °C high-temperature stress, but declined to the 0 h control level at 24 h. Therefore, under high-temperature stress, chlorophyll content in the heat-tolerant line J18-1 continued to increase, whereas that in the heat-sensitive line J190 increased only transiently and then returned to the initial level, suggesting a difference between the two lines in their ability to maintain chlorophyll homeostasis.

We measured the activities of antioxidant enzymes, including SOD (Figure 2C), POD (Figure 2D), and CAT (Figure 2E), as well as MDA (Figure 2F) content in the heat-tolerant line J18-1 and the heat-sensitive line J190 under high-temperature stress at 0 h, 6 h, and 24 h. In the heat-tolerant line J18-1, SOD, POD, and CAT activities all increased sharply and peaked after 6 h of high-temperature stress. Although these activities decreased slightly at 24 h, the antioxidant enzyme activities remained at elevated levels. This dynamic pattern is consistent with the typical antioxidant response to high-temperature stress observed in heat-tolerant plants. MDA, the end product of lipid peroxidation, serves as a direct indicator of the extent of cell membrane damage. In J190, MDA content increased sharply at 6 h and remained elevated at 24 h. In contrast, MDA content in J18-1 showed a mild increase.

### 3.3. RNA-Seq Data Quality Assessment

To elucidate the relationship between heat tolerance phenotypes and gene regulation in pepper, functional leaves were collected separately from heat-tolerant and heat-sensitive lines for transcriptome sequencing, and the comparison groups were established as described in Table 2. RNA-seq libraries were constructed from 18 samples, and the quality of the sequencing data was evaluated (Table 3). Clean reads were mapped to the *Capsicum annuum* reference genome at rates of 93.37–95.34%, of which approximately 62.57% were mapped to exonic regions. Pearson correlation coefficients among biological replicates ranged from 0.84 to 0.98, indicating acceptable reproducibility. These data indicate the reliability of the transcriptome results for subsequent analyses. All raw sequencing data are available in the NCBI SRA under BioProject accession number PRJNA1290848.

### 3.4. Differentially Expressed Genes (DEG) Analysis

**Figure 3:**
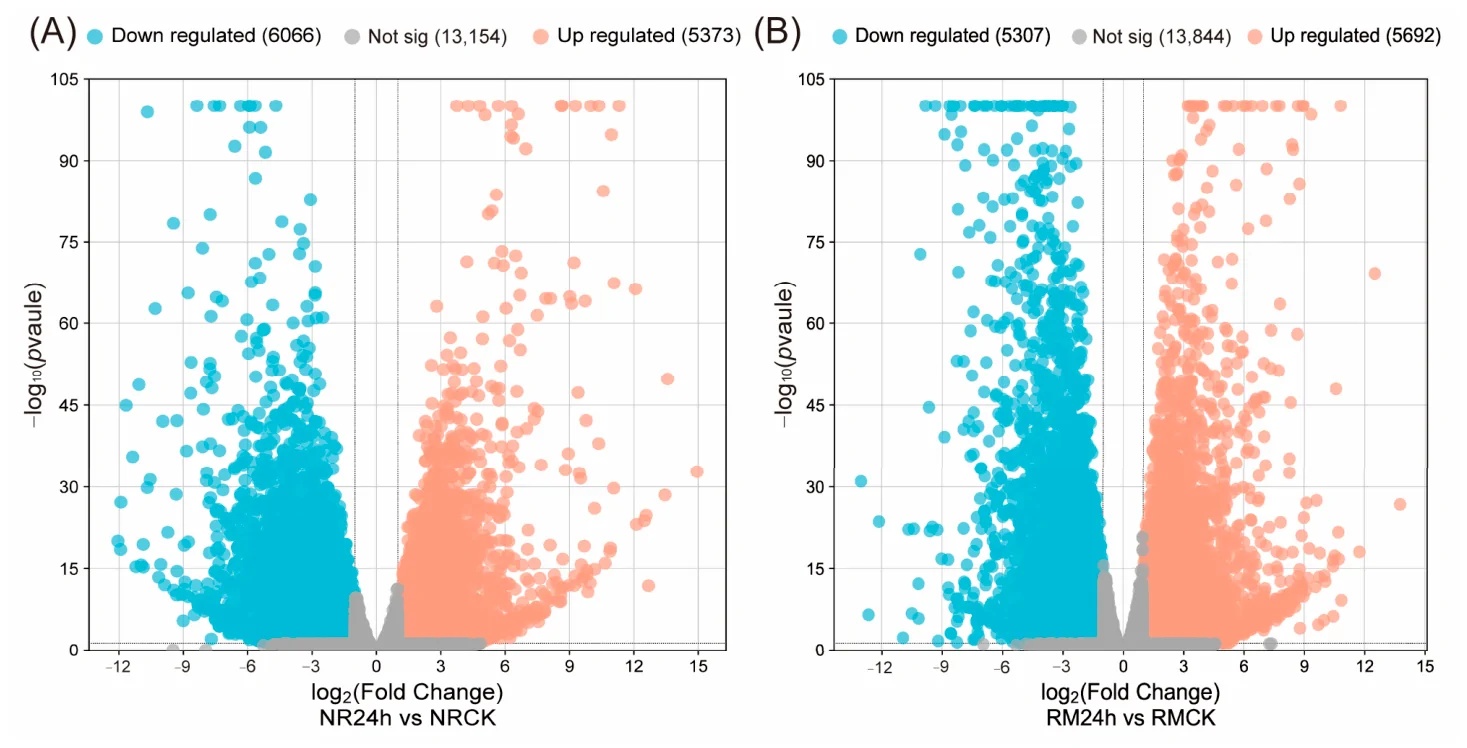
Volcano plots of DEGs under high-temperature stress. (**A**) NR24 h vs. CK volcano plots of DEGs under high-temperature stress. (**B**) RM24 h vs. RMCK volcano plots of DEGs under high-temperature stress.

Differentially expressed genes (DEGs) were defined with FDR-adjusted *p*-value < 0.05 and |log_2_ fold change| ≥ 2. In total, 16,912 unique DEGs were identified between the heat-tolerant and heat-sensitive pepper lines (Table 4). In the heat-tolerant line, 3903 DEGs were up-regulated and 4735 were down-regulated when comparing the 6 h heat-treated group with the control (NRCK). For the NR24 h group compared with CK (Figure 3A), 5373 DEGs were up-regulated and 6066 were down-regulated. In the heat-sensitive line, comparison of the RM6 h with CK showed 4014 up-regulated and 4045 down-regulated DEGs, while the comparison of the RM24 h with CK had 5692 up-regulated and 5307 down-regulated DEGs (Figure 3B).

### 3.5. GO Enrichment of DEGs

**Figure 4:**
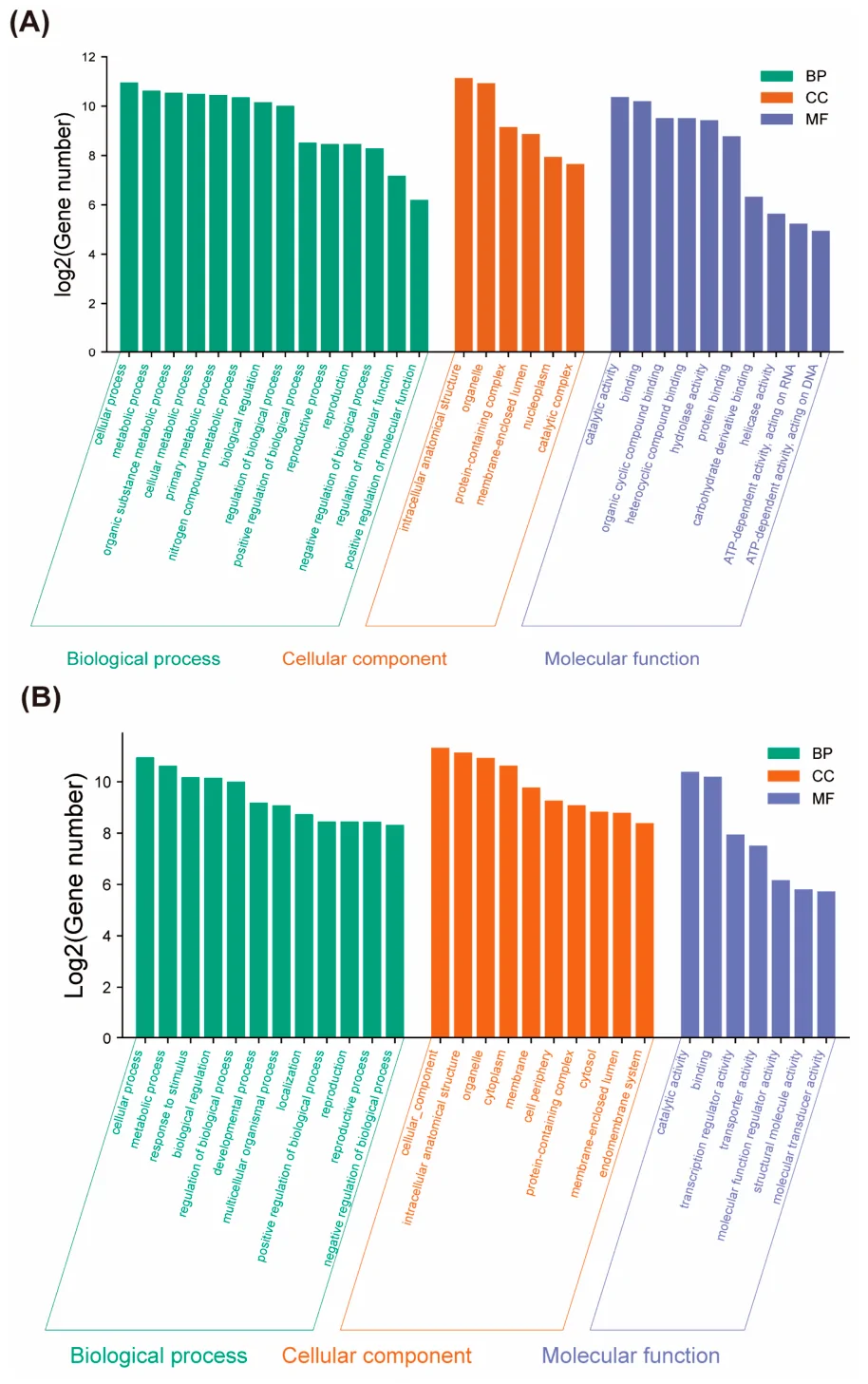
Gene ontology (GO) analysis of DEGs under high-temperature stress. (**A**) NR24 h vs. NRCK. (**B**) RM24 h vs. CK.

To explore the biological functions of DEGs under high-temperature stress, Gene Ontology (GO) enrichment analysis was performed on pepper leaves from both heat-tolerant and heat-sensitive lines across different time points. In the comparison between the NR24 h group and the control (Figure 4A), the up-regulated DEGs were significantly enriched in 14 biological processes, 6 cellular components, and 10 molecular functions. The most representative biological processes included: cellular process (GO:0009987), metabolic process (GO:0008152), organic substance metabolic process (GO:0071704), cellular metabolic process (GO:0044237), primary metabolic process (GO:0044238), and nitrogen compound metabolic process (GO:0006807). For cellular components, DEGs were significantly associated with intracellular anatomical structure (GO:0005622), organelle (GO:0043226), protein-containing complex (GO:0032991), membrane-enclosed lumen (GO:0031974), nucleoplasm (GO:0005654), and catalytic complex (GO:1902494). In terms of molecular function, DEGs were predominantly enriched in catalytic activity (GO:0003824), binding (GO:0005488), hydrolase activity, acting on ester bonds (GO:0016788), heterocyclic compound binding (GO:1901363), and hydrolase activity (GO:0016787).

In the comparison between the RM24 h group and the control (Figure 4B), the up-regulated DEGs were annotated to 12 biological processes, 12 cellular components, and 12 molecular functions. Key biological processes included: cellular process (GO:0009987), metabolic process (GO:0008152), response to stimulus (GO:0050896), biological regulation (GO:0065007), regulation of biological process (GO:0050789), developmental process (GO:0032502). DEGs were predominantly enriched in Cellular component: cellular_component (GO:0005575), intracellular anatomical structure (GO:000622), organelle (GO:0043226), cytoplasm (GO:0005737), membrane (GO:0016020), and cell periphery (GO:0071944). Molecular function terms included catalytic activity (GO:0003824), binding (GO:0005488), transcription regulator activity (GO:0140110), transporter activity (GO:0005215), molecular function regulator activity (GO:0098772), structural molecule activity (GO:0005198).

### 3.6. Analysis of DEGs Related to High-Temperature Stress Response

Temperature change can alter the fluidity of the cell membrane and induce conformational changes in certain transmembrane-spanning proteins [15]. Subsequently, the transmission of thermal signals into the cell activates second messengers such as calcium ions (Ca^2+^) and reactive oxygen species (ROS), which relay the heat signal to downstream responses. Furthermore, intracellular proteins (RBGD2/4 proteins) undergo conformational changes and assemble into stress granules that enhance plant thermo- tolerance [16]. Ultimately, the heat signal is further transmitted into the nucleus, regulating the expression of a large number of high-temperature stress-related genes. This leads to the synthesis of abundant heat shock proteins, ubiquitin-related proteins, and other factors that participate in stabilizing key enzymes and supporting various physiological activities in plants under high-temperature stress conditions [17].

**Figure 5:**
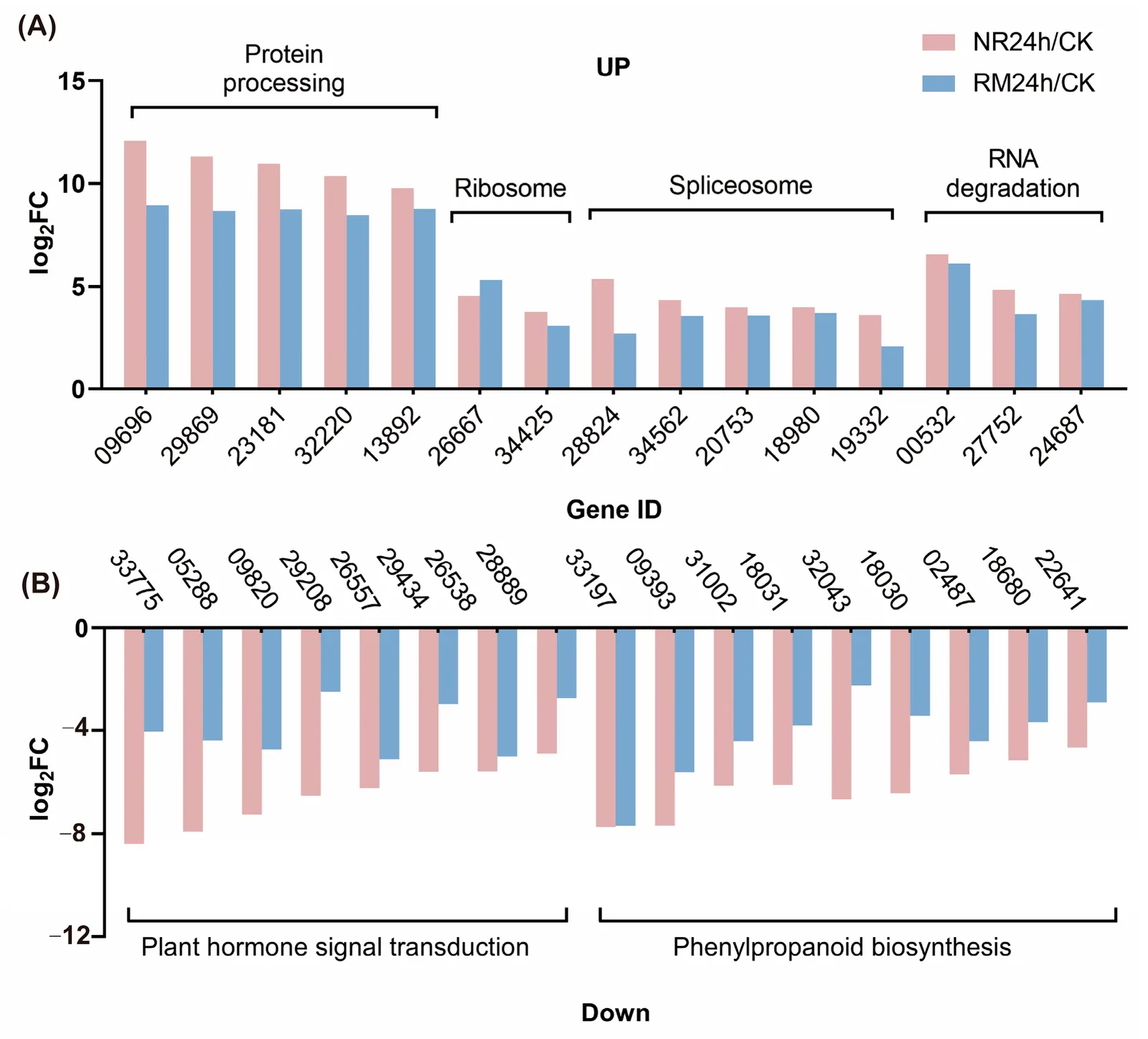
Comparison of DEGs’ fold changes at 24 h vs. CK between the heat-tolerant line J18-1 and the heat-sensitive line J190 under 42 °C high-temperature stress. (**A**) Up-regulated DEGs under high-temperature stress. (**B**) Down-regulated DEGs under high-temperature stress.

Under high-temperature treatment (Figure 5), five DEGs (09696, 29869, 23181, 32220, 13892) in Protein processing in endoplasmic reticulum, two DEGs (26667, 34425) in Ribosome biogenesis in eukaryotes, five DEGs (28824, 34562, 20753, 18980, 19332) in Spliceosome, and three DEGs (00532, 27752, 24687) in RNA degradation were significantly up-regulated (Figure 5A). Among them, NR24 h vs. NRCK (09696, 29869), RM24 h vs. RMCK (00532) exhibited high fold changes (12.07, 11.3, and 7.65). These results indicate that protein processing in the endoplasmic reticulum and RNA degradation may maintain pepper growth under high-temperature stress.

### 3.7. KEGG Pathway Enrichment of DEGs

**Figure 6:**
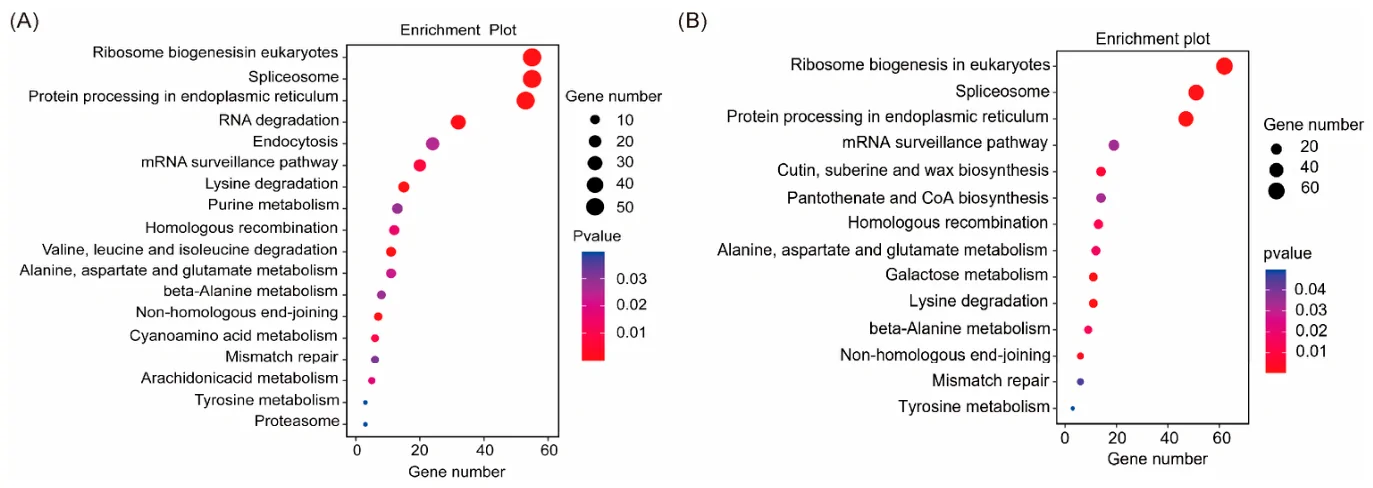
KEGG enrichment plots of differentially expressed genes in two pepper lines under high-temperature stress for 24 h. (**A**) Up-regulated DEGs in NR24 h vs. NRCK. (**B**) Up-regulated DEGs in RM24 h vs. CK.

To identify the biological pathways involved in pepper’s response to high temperature, KEGG enrichment analysis was performed using KOBAS v3.0. The transcriptomic results from pepper leaves across different treatments showed that the number of pathways enriched by up-regulated DEGs was generally comparable to that enriched by down-regulated DEGs. Taking the comparison between the heat-tolerant group at 24 h vs. the control (CK) as an example (Figure 6A), 18 and 20 pathways were enriched for up-regulated and down-regulated DEGs, respectively. The up-regulated DEGs were primarily enriched in protein processing in the endoplasmic reticulum, spliceosome, and ribosome biogenesis in eukaryotes. Among these, the protein processing in endoplasmic reticulum pathway included genes such as Small heat shock protein, chloroplastic (09696), class I heat shock protein (23181), 22.0 kDa class IV heat shock protein (13892, 29869), and 26.5 kDa heat shock protein, mitochondrial (01627). The Spliceosome pathway comprised genes including pre-mRNA-processing factor 40 (28824), small nuclear ribonucleoprotein PRP31 (20753), and 116 kDa U5 small nuclear ribonucleoprotein component (18980). The Ribosome biogenesis in eukaryotes pathway involved genes such as Cell division control protein 48-like protein C (26667, 34425), U3 small nucleolar RNA-associated protein 22 (06329), and ribonuclease P/MRP protein subunit POP1 (29630). In contrast, in the comparison between the RM24 h group and the control (RM24 h vs. RMCK; Figure 6B), the up-regulated DEGs were also primarily enriched in the ribosome biogenesis in eukaryotes, spliceosome, and protein processing in the endoplasmic reticulum.

Conversely, taking the heat-tolerant line comparison (RM24 h vs. RMCK) as an example, the down-regulated DEGs in leaves were enriched in pathways related to plant hormone signal transduction (Figure 5B). These included putative auxin-induced SAUR-like protein (33775), two-component response regulator ORR10 (05288), and putative bidirectional sugar transporter (09820). Other down-regulated pathways comprised phenylpropanoid biosynthesis, involving genes such as hypothetical protein BC332_23133 (33197), peroxidase 4 (09393), and peroxidase 21 (31688).

### 3.8. Up-Regulation of DEGs Associated with Protein Processing in the Endoplasmic Reticulum Under High-Temperature Stress

**Figure 7:**
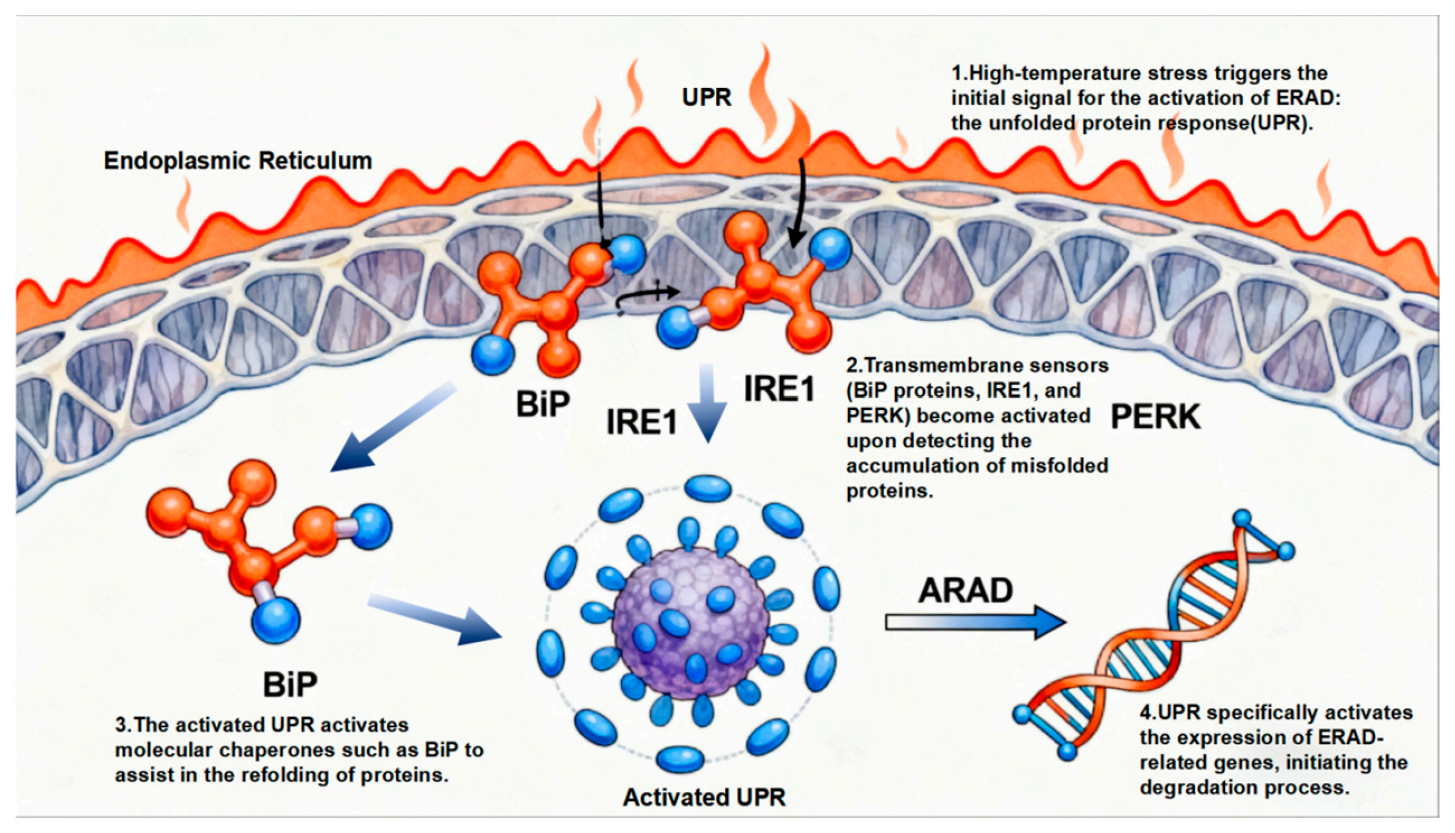
Schematic diagram of the unfolded protein response (UPR) mechanism in pepper under high-temperature stress.

KEGG pathway analysis revealed that under high temperature stress, the protein processing in endoplasmic reticulum pathway exhibited the highest enrichment level in pepper leaves, with most DEGs showing significant up-regulation. This pathway may serve as a mechanism for maintaining protein homeostasis in the endoplasmic reticulum (ER), being responsible for the synthesis, folding, modification, quality control of nascent proteins, and the degradation of misfolded proteins [18,19] (Figure 7). According to previous studies, high temperature disrupts proper protein folding within the ER of pepper, leading to the accumulation of unfolded/misfolded proteins, which directly triggers the unfolded protein response (UPR) and initiates the downstream ER-associated degradation (ERAD) pathway [20]. Subsequently, ER transmembrane sensors (BiP, IRE1, PERK) detect the buildup of unfolded proteins and switch to an activated state [21]. The activated UPR, on the one hand, up-regulates molecular chaperones to assist in protein refolding; on the other hand, if refolding fails, it specifically induces the expression of ERAD-related genes to initiate the degradation process [22,23].

**Figure 8:**
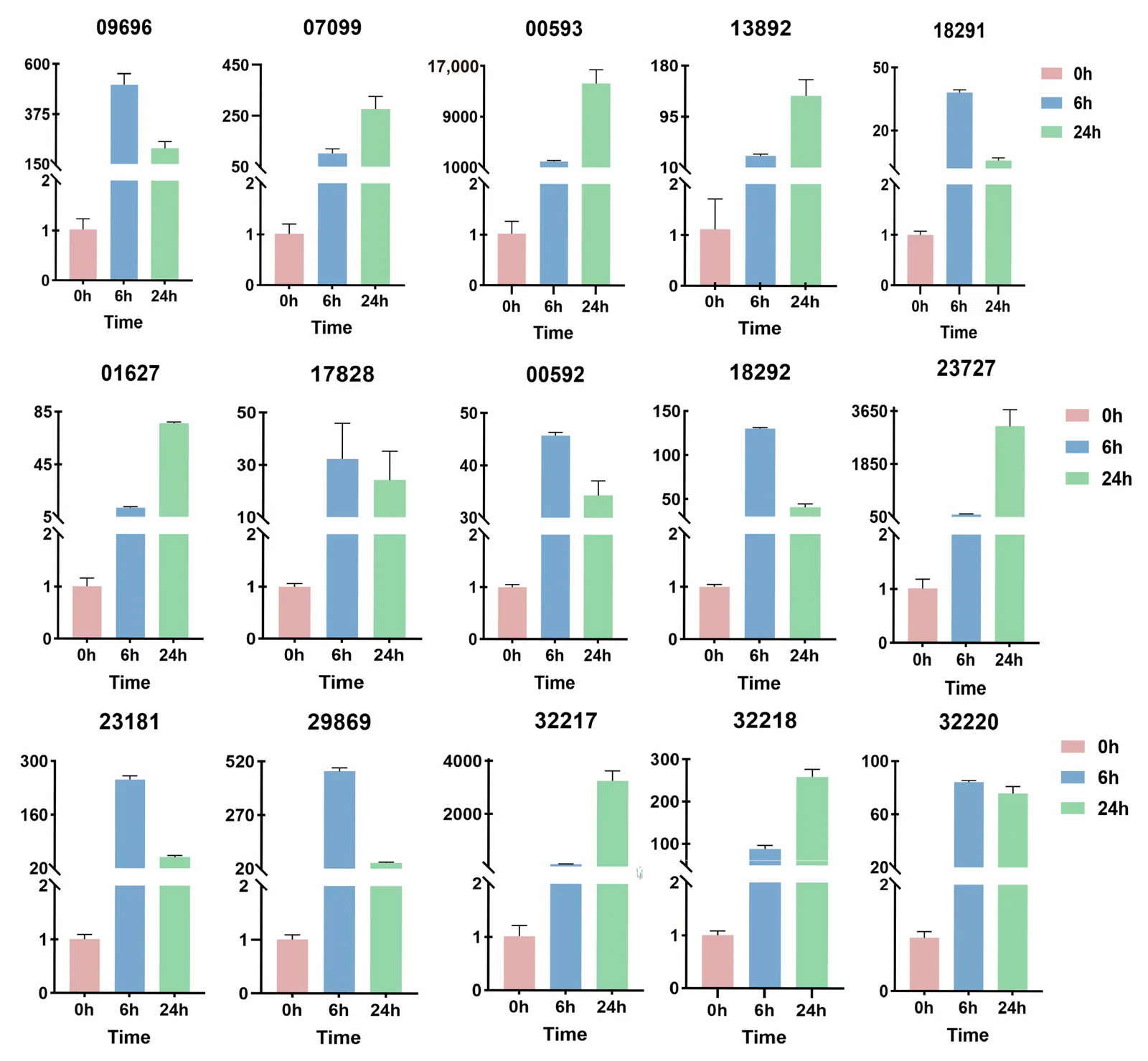
Heat-tolerant line J18-1 expression analysis of *HSP20* family genes under 42 °C for different durations. The error bars represent the standard deviation of the three biological replicates.

Notably, HSP20 family proteins exhibited fold changes within the protein processing in the endoplasmic reticulum pathway (Table 5). The small heat shock proteins (sHSPs) encoded by *HSP20* family genes are generally recognized as molecular chaperones [24,25]. According to previous studies, *HSP20* family genes respond to high-temperature stress primarily through the following three mechanisms. First, maintaining protein homeostasis: high temperatures lead to protein misfolding or denaturation [26]. As molecular chaperones, HSP20 proteins can bind to these misfolded proteins, prevent their irreversible aggregation, and assist in restoring their correct conformation, thereby preserving intracellular protein homeostasis [27]. For example, the *CaHsp25.9* gene in pepper may be induced by high-temperature stress [28]. Silencing this gene increases reactive oxygen species (ROS) accumulation under high-temperature, salt, and drought stress conditions, whereas its overexpression reduces ROS accumulation and enhances antioxidant enzyme activity, indicating the vital role of this gene in maintaining intracellular stability. Second, participating in signal transduction: HSP20 proteins may be involved in the signal transduction pathways that enable pepper to respond to high-temperature stress, thereby influencing the expression of genes related to ER-associated degradation (ERAD). Studies have shown that heat shock transcription factors (HSFs) play key roles in the high-temperature stress response by binding to heat shock elements (HSEs) and activating the expression of high-temperature responsive genes, including *HSP20* family genes [29,30]. Third, *HSP20* family genes may strengthen the antioxidant capacity of pepper. For instance, overexpression of *CaHsp16.4* could stabilize the ROS-scavenging system, thereby enhancing tolerance to high-temperature stress and drought stress and reducing oxidative damage to cells [31]. This helps maintain normal endoplasmic reticulum function and ensures the proper progression of the ERAD pathway. Based on previous studies, *HSP20* family genes may be associated with heat-tolerance phenotypes at the seedling stage of pepper. Therefore, we performed qRT-PCR analysis of the *HSP20* family genes identified from the transcriptome results (Figure 8).

### 3.9. Expression of Differentially Expressed Genes (DEGs) Involved in Plant Hormone Signal Transduction Under High-Temperature Stress

**Figure 9:**
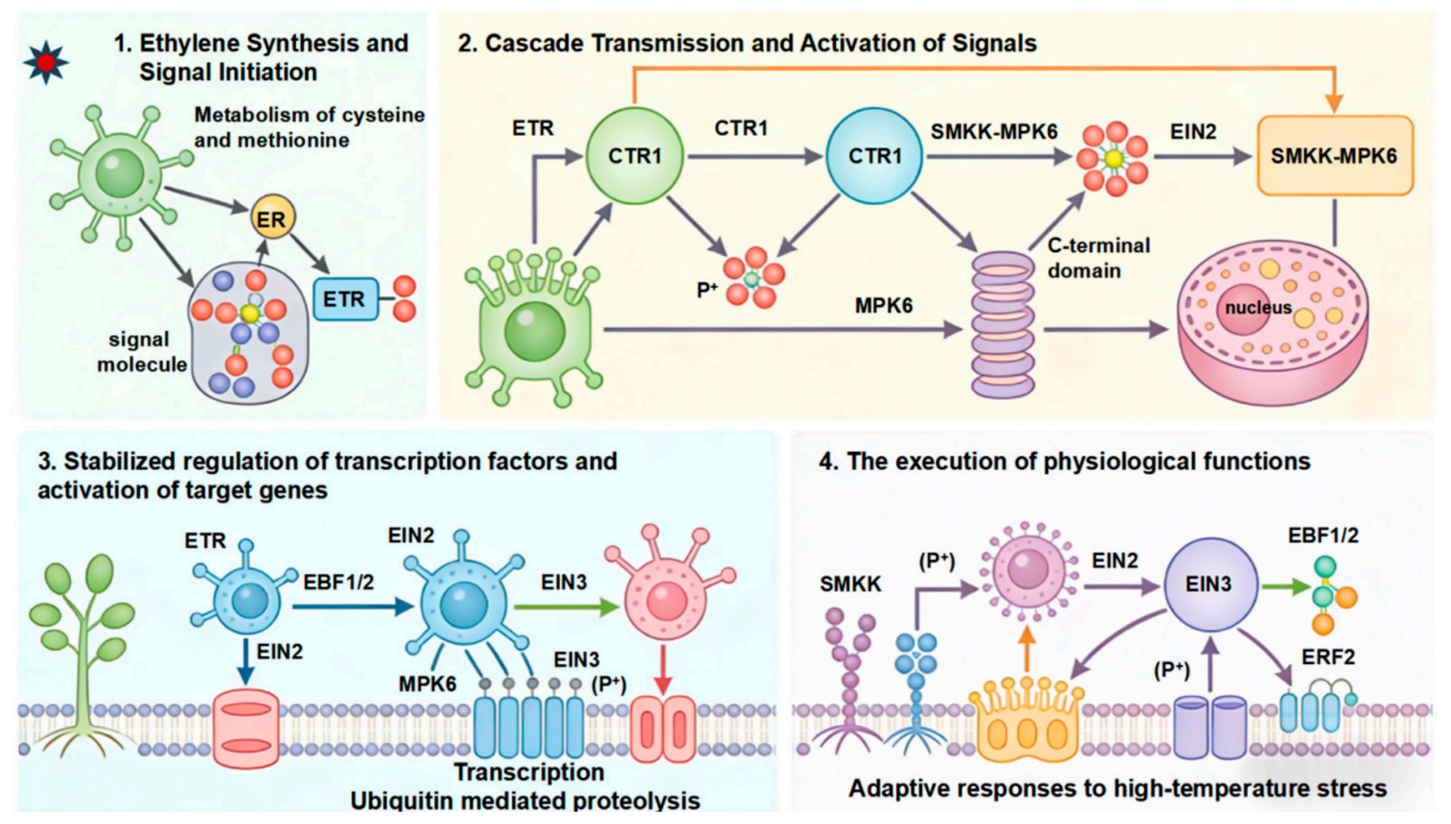
Working Mechanism Diagram of Ethylene-Mediated Plant Response Pathway to High-Temperature Stress.

KEGG pathway analysis also indicated that, under high-temperature stress, plant hormone signal transduction was among the significantly enriched pathways in pepper leaves. Based on current literature, the ethylene-mediated response of plants to high-temperature stress is proposed to proceed through the following steps (Figure 9). Notably, ERF family genes also showed relatively high fold changes in this pathway (Table 6). High-temperature stress may promote ethylene synthesis by regulating cysteine and methionine metabolism [32]. Ethylene diffuses as a signaling molecule to the endoplasmic reticulum (ER), binds to ethylene receptors (ETRs) on the ER membrane, and can initiate downstream signal transduction [33,34]. Under non-stress conditions, ETR interacts with CTR1, which helps maintain CTR1 activity and suppress downstream signaling [35]. Upon ethylene binding to ETR, the inhibitory effect of CTR1 is thought to be relieved, thereby permitting activation of the SIMKK (MAPKK)–MPK6 (MAPK) kinase cascade [36,37,38]. MPK6 can phosphorylate and activate EIN2, leading to the release of the C-terminal domain of EIN2 from the ER and its translocation into the nucleus, where it may initiate downstream transcriptional regulation [39]. After entering the nucleus, EIN2 may promote the accumulation of EIN3 through two mechanisms: on the one hand, EIN2 may inhibit EBF1/2-mediated ubiquitination and degradation, thereby contributing to the stabilization of EIN3 protein; on the other hand, EIN3 can bind to the promoter of ERF2 and activate its transcription [40,41]. Acting as a downstream transcription factor, ERF2 may further bind to ethylene-responsive elements (GCC-box) in target genes, potentially initiating the expression of high-temperature stress-related genes [42]. Ultimately, the expression of target genes activated through this signaling cascade may contribute to an adaptive response to high-temperature stress. In addition, this pathway may participate in regulating physiological processes related to fruit ripening and senescence, potentially coordinating the balance between growth and stress responses under elevated temperatures [43,44]. In this proposed model, a feature of this pathway is the conversion of the ethylene signal into gene expression regulation. Through cascaded signal transmission, homeostatic control of transcription factors, and other steps, this pathway may integrate high-temperature stress signals and initiate adaptive physiological and metabolic adjustments, thereby potentially contributing to plant thermotolerance.

### 3.10. qPCR Validation of RNA-Seq Identified DEGs

**Figure 10:**
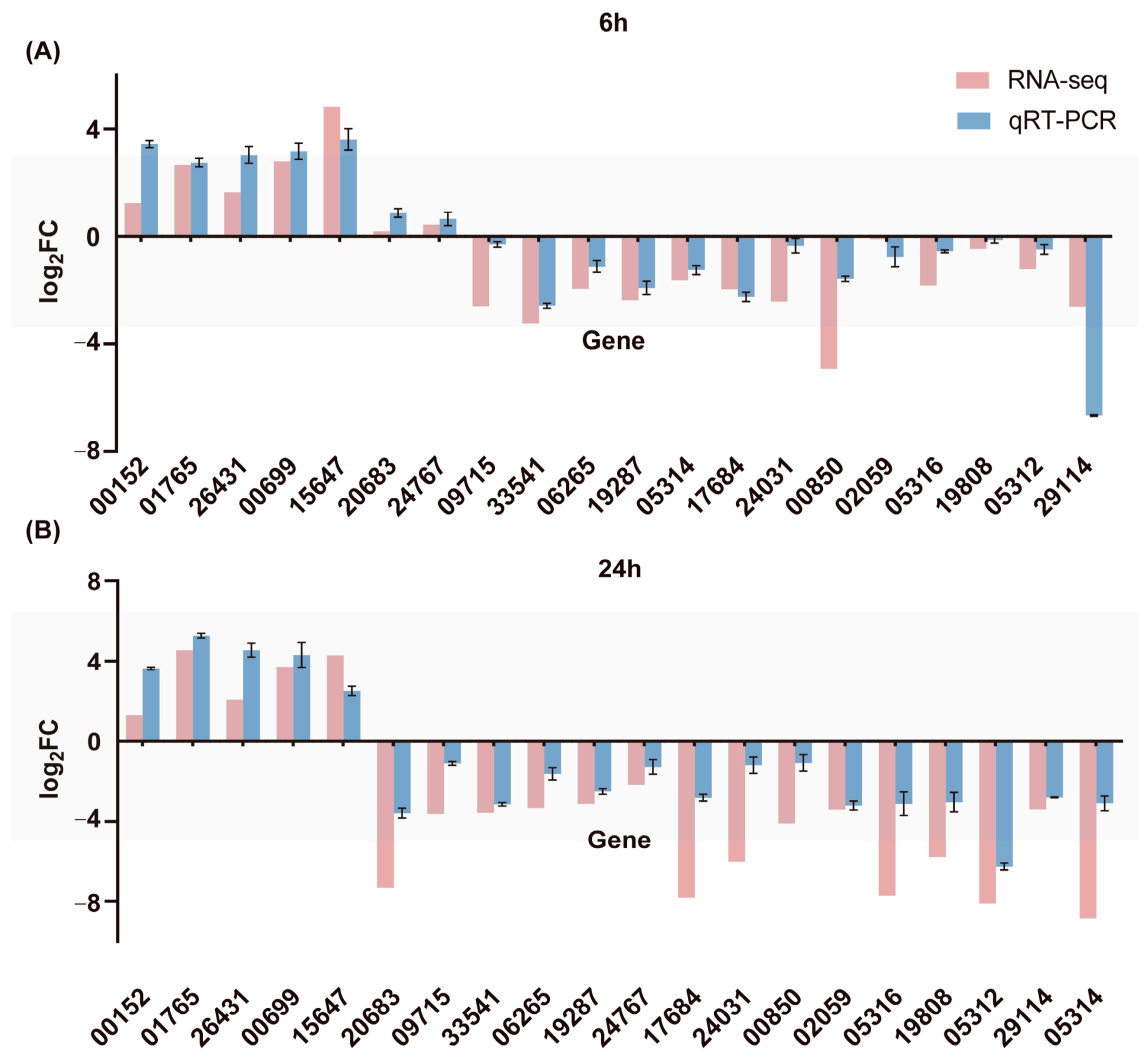
qPCR validation of partially enriched DEGs in pepper leaves under high-temperature stress. (**A**) Validation of RNA-seq expression results by qRT-PCR at 6 h. (**B**) Validation of RNA-seq expression results by qRT-PCR at 24 h. The error bars in represent the standard deviation of the three biological replicates.

To validate the RNA-Seq results, 20 DEGs were selected based on their differential expression levels and biological relevance for qPCR verification at 6 h and 24 h (Figure 10). These genes included those involved in flavonoid biosynthesis (09715, 33541, and 06265), plant hormone signal transduction (00152, 19287), and photosynthesis–antenna proteins (05314, 17684, 24031), among others. qPCR analysis confirmed that the expression patterns of all selected genes were highly consistent with those observed in the RNA-Seq dataset, supporting the reliability and accuracy of the transcriptomic analysis.

## 4. Discussion

According to transcriptomic profiles, annotated functions of DEGs, reported knowledge of candidate heat-responsive pathways, and selected physiological indices including chlorophyll, SOD, POD, CAT, and MDA, physiological measurements revealed distinct phenotypic differences between the heat-tolerant line J18-1 and the heat-sensitive line J190 under short-term high-temperature stress. At 6 h of high-temperature stress, chlorophyll content increased moderately in J18-1 and increased significantly in J190. By 24 h, chlorophyll content continued to increase in J18-1, whereas the chlorophyll level of J190 declined back to the control level. The dynamic changes in chlorophyll content suggest that J18-1 may maintain chlorophyll homeostasis under high-temperature stress. This trait may be attributed to the stable function of chlorophyll-biosynthesis-related enzymes such as δ-aminolevulinic acid synthase and protochlorophyllide reductase, which are less susceptible to heat-induced denaturation at 42 °C [45,46]. Moreover, the active antioxidant system in J18-1 may mitigate ROS-triggered injury to chloroplast membranes, potentially maintaining thylakoid integrity and reducing chlorophyll degradation [47]. In contrast, prolonged high-temperature stress may denature and inactivate chlorophyll-synthetic enzymes in J190, potentially blocking chlorophyll biosynthesis and impairing photosynthetic capacity [48]. The transient chlorophyll elevation observed in pepper seedlings at the early high-temperature stage is speculated to serve as a compensatory strategy for compromised photosynthesis; J18-1 may exhibit a controlled emergency response, whereas J190 may fail to sustain chlorophyll maintenance under short-term high-temperature stress.

We also detected clear genotype-specific differences in antioxidant enzyme activities and MDA accumulation. In J18-1, SOD, POD, and CAT activities rapidly increased at the early stress stage to scavenge excess ROS, followed by a slight decline in activities at 24 h, potentially representing a homeostatic transition of the antioxidant system [49]. MDA content remained consistently low in J18-1 and was only slightly elevated at 24 h, suggesting an intact cell membrane structure [50]. In contrast, J190 showed weak and transient antioxidant enzyme responses: SOD, CAT, and POD activities only slightly increased at 6 h and fell below the control level at 24 h. Meanwhile, MDA content increased sharply at 6 h and continued to rise at 24 h, suggesting severe membrane lipid peroxidation. Such defects in J190 may originate from insufficient heat-induced transcriptional activation of antioxidant enzyme genes, alongside heat-induced structural denaturation of antioxidant proteins, which together may weaken ROS-scavenging capacity and aggravate membrane damage. The heat-tolerant line J18-1 may establish an efficient and persistent antioxidant system accompanied by limited membrane peroxidation damage, which may constitute its core physiological foundation for heat tolerance. By comparison, inadequate antioxidant activation and progressive membrane injury under short-term high-temperature stress may be responsible for the heat sensitivity of J190.

The transcriptomic results revealed by KEGG pathway enrichment were intrinsically associated with the physiological response characteristics of pepper leaves under short-term high-temperature stress. The protein processing in the endoplasmic reticulum pathway was markedly activated, accompanied by the up-regulation of multiple heat shock protein-encoding genes. These up-regulated genes may promote the remodeling of denatured proteins and limit excessive accumulation of reactive oxygen species (ROS), thereby potentially providing a molecular explanation for the dynamic changes in SOD, POD, and CAT antioxidant enzyme activities and in the content of malondialdehyde (MDA), a marker of membrane lipid peroxidation [51,52]. In contrast, the plant hormone signal transduction pathway was suppressed, in which the transcription of auxin-related SAUR family genes was down-regulated (33775); this may contribute to the chlorophyll degradation process and is consistent with the physiological phenotype of reduced chlorophyll content in leaves [53]. The transcriptional suppression of the phenylpropanoid biosynthesis pathway and the expression fluctuations of peroxidase-encoding genes within this pathway were mutually corroborated by changes in POD enzyme activity (09393, 31688), suggesting that transcriptional regulation may participate in the remodeling of cellular antioxidant homeostasis [54]. The up-regulation of the spliceosome pathway and the ribosome biogenesis in eukaryotes pathway may provide a molecular basis for de novo translation of stress-responsive proteins, thereby potentially coordinating the physiological adaptive regulation of pepper seedlings under short-term high-temperature stress.

KEGG enrichment results were similar between the two pepper lines, suggesting that the pathways responding to short-term high-temperature stress may be conserved in pepper seedlings. Nevertheless, genes within these pathways showed divergent fold changes, suggesting that the difference in heat tolerance between the two lines may arise mainly from the varied magnitude of gene responses within these conserved pathways. High-temperature stress may lead to the accumulation of misfolded proteins in the endoplasmic reticulum (ER) and may simultaneously trigger the heat shock response (HSR) in the cytoplasm and the unfolded protein response (UPR) in the ER [55]. Studies have suggested that excessive misfolded proteins may be further degraded via ER-associated degradation (ERAD) [56]. This process is generally considered to involve the following steps. First, in protein targeting and translocation into the ER, nascent polypeptide chains, guided by signal peptides and assisted by the signal recognition particle (SRP), bind to the Sec61 translocon complex on the ER membrane and are translocated across the membrane into the ER lumen (soluble proteins) or anchored into the ER membrane (membrane proteins). Second, in protein folding and post-translational modifications, molecular chaperones such as BiP/GRP78 bind to unfolded proteins, preventing their aggregation and promoting proper folding [57]. Protein disulfide isomerase (PDI) catalyzes the formation of disulfide bonds to stabilize protein tertiary structures [58]. N-glycosylation, the covalent attachment of core oligosaccharide moieties to asparagine residues, and hydroxylation are considered key post-translational modifications that may modulate the stability and functional properties of proteins [59]. Third, in terms of protein quality control and degradation, misfolded or improperly modified proteins may undergo ERAD. They are thought to be retrotranslocated to the cytosol and degraded by the ubiquitin-proteasome system. When the accumulation of misfolded proteins exceeds the processing capacity of the ER, the UPR may be activated [60]. Through the three main signaling pathways PERK, IRE1, and ATF6, the UPR may down-regulate global protein synthesis, up-regulate chaperone expression, and enhance ERAD activity to restore ER homeostasis [61,62]. In this study, the several-fold up-regulation of HSP20 family genes may suggest a possible indirect influence of these genes on the ERAD pathway by strengthening the antioxidant capacity of pepper.

High-temperature stress may promote ethylene (ETH) synthesis. Through the ETR–EIN2–EIN3 pathway, ETH may regulate the expression of ERF genes and modulate the progression of senescence to potentially balance stress defense and the maintenance of leaf function. Compared with previous studies, the *ERF* family genes may respond to high-temperature stress through the following two pathways. On one hand, *ERF* family genes act by binding to specific cis-elements to activate the transcription of heat-responsive genes. Proteins encoded by *ERF* family genes contain a conserved *ERF* domain that can specifically bind to the ethylene-responsive element (ERE, GCC-box) in the promoter regions of downstream genes [63]. Ethylene acts as a signaling molecule to induce the expression of certain ERF genes in pepper. Studies have shown that overexpression of the GCC-box-binding *CaERF* gene in pepper can significantly up-regulate a series of defense-related genes in *Arabidopsis*, thereby enhancing its heat tolerance. On the other hand, they can act as signaling nodes that link ethylene with multiple hormone response pathways. *ERF* family genes often serve as crosstalk nodes between different hormone signaling pathways, coordinating ethylene and other hormonal signals to collectively respond to high-temperature stress [64]. For example, *ZmERF1* transgenic *Arabidopsis* lines showed higher salt tolerance, drought resistance, and heat resistance. There were many cis-regulatory elements related to stress responses in the *ZmERF1* promoter sequence. It may be induced by ABA and ethylene treatment in maize, suggesting that it might be at the crossroads of multiple hormone signaling pathways [65]. In high-temperature environments, such cross-regulation integrates stress-response signals from different hormones, creating a synergistic effect. Moreover, the promoters of most *CaAP2/ERF* genes in the pepper genome contain response elements for multiple hormones, such as abscisic acid and jasmonate, further indicating that *ERF* genes can mediate crosstalk between ethylene and other hormones, thereby refining the regulatory network for high-temperature stress response [66]. Certain *ERF* genes may participate in the regulation of genes involved in the synthesis of osmoregulatory substances (proline and betaine). These osmolytes help maintain cellular osmotic pressure under high temperature stress, protect cell structure stability, reduce water loss, and improve plant water-retention capacity, thereby alleviating the combined stress imposed by high temperature and drought. Finally, some *ERFs* maintain gene-expression homeostasis to adapt to high-temperature stress. Some *ERF* genes maintain physiological homeostasis in pepper under high-temperature through reverse regulation of their expression levels. For example, the *CaERF70* gene in pepper, which belongs to the ERF-VII group and exhibits transcriptional activation activity, is significantly suppressed under high-temperature conditions [67]. It is hypothesized that down-regulation of *CaERF70* expression may release the activation of certain negatively regulated stress-responsive genes, avoiding unnecessary energy consumption. This allows pepper to concentrate energy on activating key heat-response pathways. Such an expression regulatory pattern represents another important way by which *ERF* family genes adapt to short-term high-temperature stress.

## 5. Conclusions

In summary, this study identified the heat-tolerant line J18-1 and heat-sensitive line J190 from 21 pepper accessions of the “J-series”. After exposure to 42 °C for 48 h, physiological and biochemical indices of these two lines exhibiting distinct heat tolerance characteristics were measured. Based on transcriptomic analysis of leaves collected at 0 h, 6 h, and 24 h of heat treatment, time-dependent DEGs were identified. The candidate genes identified here may expand the list of heat-responsive genes in pepper. KEGG analysis revealed that protein processing in the endoplasmic reticulum and plant hormone signal transduction were the two most critical pathways involved in pepper response to high-temperature stress. Within these pathways, the HSP20 family protein and *ERF* family genes, respectively, displayed fold changes. This work supplies both theoretical support and genetic resources for breeding heat-resistant pepper varieties. Future studies may focus on functional validation of candidate genes and further exploration of gene–gene interactions and regulatory networks, thereby offering more precise technical support for improving heat tolerance in *Capsicum annuum* L.

## Figures and Tables

**Table 1:** Heat injury index rating after high-temperature stress on peppers.

No.	Cultivar	Total Plants	Grade 0	Grade 1	Grade 2	Grade 3	Grade 4	Heat Injury Index (%)
1	J1-1	8			4	3	1	65.6
2	J9-2	8				2	6	93.8
3	J19-1	8			2	4	2	75
4	J19-3	8			1	6	1	75
5	J21	8	5	3				9.4
6	J59	8				1	7	96.9
7	J102	8				5	3	84.4
8	J163	8			4	3	1	65.6
9	J166-1	8			1	2	5	90.6
10	J187	8				1	7	96.9
11	J189	8			1		7	93.8
12	J190	8					8	100
13	J81	8			2	3	2	65.6
14	J18-1	8	6	2				6.25
15	J43-4	8		2		2	4	75
16	J75-1	8			5	3		59.4
17	J140	8			5	2	1	62.5
18	J176	8		3		2	3	65.6
19	J188	8				4	4	87.5
20	J195-1	8				3	5	90.6
21	J205	8			2	3	3	78.1

**Table 2:** Settings for comparative analysis of transcriptome groups.

Comparative Analysis	Treatment	Control
Group 1	NR6 h vs. NRCK	
Group 2	NR24 h vs. NRCK	
Group 3	RM6 h vs. RMCK	
Group 4	RM24 h vs. RMCK	

**Table 3:** Summary statistics of RNA-seq data.

Sample	Raw Reads	Clean Reads	Mapped Reads	Mapped (%)	Unique Mapped Reads	Clean Q30 (%)	Exon (%)
NRCK1	80,068,042	79,157,766	75,253,591	95.07%	70,317,499	97.49%	69.30%
NRCK2	80,003,768	79,064,174	74,573,531	94.32%	69,587,135	97.26%	70.03%
NRCK3	80,054,086	79,117,968	75,381,413	95.28%	72,225,051	97.36%	65.81%
NR6 h1	78,043,876	77,175,174	73,142,387	94.77%	69,070,435	97.54%	60.17%
NR6 h2	80,066,802	79,075,136	74,289,727	93.95%	69,899,669	97.08%	61.50%
NR6 h3	80,078,944	79,156,472	74,419,643	94.02%	70,681,480	97.55%	60.18%
NR24 h1	78,356,574	77,433,304	72,789,857	94.00%	70,285,095	97.48%	55.45%
NR24 h2	80,076,974	79,116,028	74,082,080	93.64%	71,439,152	97.38%	54.49%
NR24 h3	80,058,160	79,088,552	74,970,801	94.79%	72,148,234	97.83%	56.47%
RMCK1	80,046,670	79,099,674	75,326,980	95.23%	70,809,823	97.90%	69.11%
RMCK2	80,082,308	79,113,082	75,429,573	95.34%	70,699,891	97.92%	69.56%
RMCK3	77,000,336	76,044,102	72,499,044	95.34%	67,894,773	97.87%	70.24%
RM6 h1	80,052,276	79,004,942	73,960,120	93.61%	70,090,011	97.73%	63.01%
RM6 h2	80,050,708	78,962,680	73,729,283	93.37%	69,759,313	97.60%	63.56%
RM6 h3	80,033,652	78,986,610	74,103,314	93.82%	69,694,656	97.76%	62.02%
RM24 h1	80,026,292	78,983,296	74,563,946	94.40%	71,361,430	97.84%	56.33%
RM24 h2	80,093,268	79,053,744	74,116,874	93.76%	70,971,690	97.76%	58.73%
RM24 h3	80,000,442	78,949,974	74,064,881	93.81%	71,172,678	97.72%	60.29%

NRCK: heat-tolerant line J18-1 under 42 °C for 0 h. NR6 h: heat-tolerant line J18-1 under 42 °C for 6 h. NR24 h: heat-tolerant line J18-1 under 42 °C for 24 h. RMCK: heat-sensitive line J190 under 42 °C for 0 h. RM6 h: heat-sensitive line J190 under 42 °C for 6 h. RM24 h: heat-sensitive line J190 under 42 °C for 24 h.

**Table 4:** Summary of differentially expressed genes.

Group	UP	Down	Total
NR6 h vs. NRCK	3903	4735	8638
NR24 h vs. NRCK	5373	6066	11,439
RM6 h vs. RMCK	4014	4045	8059
RM24 h vs. RMCK	5692	5307	10,999
Total Unique DEGs	16,912		

**Table 5:** Pepper HSP20 family protein DEGs involved in high-temperature stress response.

Gene ID (NR24h/CK)	Log_2_FC	*p*-adj	Gene Description
T459_09696	12.08	2.22 × 10^−64^	Small heat shock protein, chloroplastic
T459_29869	11.31	1.88 × 10^−142^	22.0 kDa class IV heat shock protein
T459_23181	10.95	1.86 × 10^−92^	class I heat shock protein
T459_32220	10.37	9.77 × 10^−103^	18.2 kDa class I heat shock protein
T459_13892	9.73	7.97 × 10^−41^	22.0 kDa class IV heat shock protein
T459_01627	9.73	3.21 × 10^−62^	26.5 kDa heat shock protein, mitochondrial
T459_07099	9.28	3.88 × 10^−188^	16.9 kDa class I heat shock protein 3
T459_00593	8.62	6.60 × 10^−163^	Heat shock 22 kDa protein, mitochondrial
T459_18292	7.34	6.14 × 10^−43^	hypothetical protein
T459_00592	6.96	5.60 × 10^−90^	Heat shock 22 kDa protein, mitochondrial
T459_17828	6.89	4.12 × 10^−6^	23.2 kDa heat shock protein
T459_23727	6.69	2.98 × 10^−63^	Heat shock 22 kDa protein, mitochondrial
T459_32218	6.49	1.71 × 10^−70^	17.8 kDa class I heat shock protein
T459_32217	6.35	2.34 × 10^−36^	18.2 kDa class I heat shock protein
T459_18291	5.79	1.26 × 10^−50^	hypothetical protein

**Table 6:** Pepper *ERF* gene family DEGs involved in high-temperature stress response.

Gene ID (NR24h/CK)	Log_2_FC	*p*-adj	Gene Description
T459_05107	7.40	4.24 × 10^−6^	Ethylene-responsive transcription factor ERF109
T459_12450	5.05	3.74 × 10^−19^	Ethylene-responsive transcription factor
T459_14078	4.82	0.01	Ethylene-responsive transcription factor LEP-like
T459_04175	4.78	6.62 × 10^−35^	Ethylene-responsive transcription factor
T459_00699	3.72	0.04	Ethylene-responsive transcription factor 2
T459_20141	3.41	5.25 × 10^−16^	Ethylene-responsive transcription factor
T459_14986	2.80	2.26 × 10^−6^	Ethylene-responsive transcription factor 5-like
T459_16229	−3.19	3.95 × 10^−7^	AP2-like ethylene-responsive transcription factor ANT
T459_10563	−3.29	2.29 × 10^−7^	Ethylene-responsive transcription factor ERF003
T459_10516	−4.05	1.60 × 10^−23^	Ethylene-responsive transcription factor WIN1
T459_22132	−4.53	1.99 × 10^−14^	Ethylene-responsive transcription factor WRI1
T459_03273	−4.59	1.32 × 10^−21^	Ethylene-responsive transcription factor TINY

## Data Availability

Raw RNA-seq sequencing data have been deposited in the NCBI Sequence Read Archive (SRA) under BioProject accession PRJNA1290848. The dataset is publicly accessible.

## Supplementary Materials

The following supporting information can be downloaded at: https://www.mdpi.com/article/10.3390/biology15211925/s1, Table S1: Heat injury index rating after high-temperature stress on peppers; Table S2: Heat injury index rating after high temperature stress on peppers; Table S3: qPCR Primer Design for DEGs; Table S4: qPCR Primer Design for *HSP20* Family Gene; Table S5: Abbreviations; Figure S1: PCA analysis of All samples; Figure S2: Heatmap analysis of gene expression across various samples; The qPCR results of *HSP20* family genes, the qPCR results of 20 DEGs and the determination results of physiological parameters of pepper are included in the original data file.
