# Supplementary material for: Transcriptome Analysis Reveals Candidate Genes and Regulatory Pathways Involved in Pepper Seedlings (Capsicum annuum L.) Response to Short-Term High-Temperature Stress
Source: Biology (Basel). 2026 Oct 23;15(21):1925. doi: 10.3390/biology15211925 (PMC13762258; doi:10.3390/biology15211925)
Supplement: Supplementary file 1 [file biology-15-01925-s001.zip › biology-4575529-supplementary.pdf]

Table S1. Heat injury index rating after high temperature stress on peppers.

Heat injury index rating after high temperature stress on peppers（First）								
No.	Cultivar	Total plants	Grade 0	Grade 1	Grade 2	Grade 3	Grade 4	Heat injury index (%)
1	J1-1	6			2		4	83.3
2	J9-2	6		5			1	37.5
3	J19-1	6		5		1		33.3
4	J19-3	6			2	1	3	79.1
5	J21	6	5			1		12.5
6	J59	6			3		3	75
7	J102	6		1	2		3	70.8
8	J163	6			2		4	83.3
9	J166-1	6			1	2	3	83.3
10	J187	6				2	4	91.6
11	J189	6			2	1	3	79.1
12	J190	6					6	100
13	J81	6			1	3	2	79.1
14	J18-1	6	4	2				8.3
15	J43-4	6				2	4	91.6
16	J75-1	6			3	3		62.5
17	J140	6			3	2	1	50
18	J176	6				2	4	91.6
19	J188	6				2	4	91.6
20	J195-1	6				3	3	87.5
21	J205	6				3	3	87.5

Table S2. Heat injury index rating after high temperature stress on peppers.

Heatinjuryindexratingafterhightemperaturestressonpeppers（Second）								
No.	Cultivar	Total plants	Grade 0	Grade1	Grade2	Grade3	Grade4	Heatinjuryinde x(%)
1	J1-1	8			4	3	1	65.6
2	J9-2	8				2	6	93.8
3	J19-1	8			2	4	2	75
4	J19-3	8			1	6	1	75
5	J21	8	5	3				9.4
6	J59	8				1	7	96.9
7	J102	8				5	3	84.4
8	J163	8			4	3	1	65.6
9	J166-1	8			1	2	5	90.6
10	J187	8				1	7	96.9
11	J189	8			1		7	93.8

12	J190	8					8	100
13	J81	8			2	3	2	65.6
14	J18-1	8	6	2				6.25
15	J43-4	8		2		2	4	75
16	J75-1	8			5	3		59.4
17	J140	8			5	2	1	62.5
18	J176	8		3		2	3	65.6
19	J188	8				4	4	87.5
20	J195-1	8				3	5	90.6
21	J205	8			2	3	3	78.1

Table S3. qPCR Primer Design for Differentially Expressed Genes

Gene ID	Primer
T459_09715	F′ : 5' AATGCTTAGGCTTCTCGCTACTTACTC 3'<br>R′: 5' GCTCTCCATGAGGACTTTATCTTGATTC 3'
T459_33541	F′:5' CCTGTGGAATGGTTCCGTGG 3'<br>R′:5' GGTAATTCCATCACCAACAGGAAGC 3'
T459_06265	F′:5' GAAGGGCACAACAAAGTGATAATGG 3'<br>R′:5' AGATGCTTCCCTTTCGGAGTTGAC 3'
T459_00152	F′:5' GGCACAACAAAGTGATAATGGCG 3'<br>R′:5' GCAGCCTCAACAACACTGCACTTC 3'
T459_04029	F′:5' GGTGAAGGAGGCTATTGTAGAAGTGG 3'<br>R′:5' GACAGCTCTGAGCGCGTAGAATC 3'
T459_19287	F′:5' ATGAGCTAGTGGAGTTGTTGTGGCG 3'<br>R′:5' GCATTCATTAGGATCATAGCCTGG 3'
T459_05314	F′:5' ATCCAGCCTTGAACCACACAGC 3'<br>R′:5' CCAAGAACCGAGAACTCGAGGTG 3'
T459_17684	F′:5' CCAGAGAACTTGAGATGGTTCGTG 3'<br>R′:5' CAGGGTAACCAACTTCATTAGGAGG 3'
T459_24031	F′:5' CTGAGACATTCGCTAGGAACCGTG 3'<br>R′:5' CGCCTCACCGAATGTGACACC 3'
T459_00850	F′:5' TGATGTACCTGAAGGGAATACGGAG 3'<br>R′:5' CACGAGCTTCACCGGCCATAG 3'
T459_01765	F′:5' ATGGCTTGGTGGATTCCGTTC 3'<br>R′:5' GACTCTTGCAATGCCTCGATTC 3'
T459_26431	F′:5' CCGATTCTGGTTCTCGCACTG 3'<br>R′:5' TGCGATAACTTCAGACGGCTATTC 3'
T459_24767	F′:5' GGTGGACCGTTCTTTAACCCTC 3'<br>R′:5' CCAACATAGCAAGCCTTCCATTC 3'
T459_05312	F′:5' GAACGGTAGACTTGCTATGTTCTCTATG 3'<br>R′:5' CCCAGGCGTTGTTGTTAACTGG 3'
T459_02059	F′:5' CAGTAGCACCGCCGATGACAGTG 3'<br>R′:5' TCTTCATCCAAGCCTATGTGACAGG 3'

T459_29114	F′:5' CGGTGGCTGTGAACTCCGTC 3'<br>R′:5' CGTTGAACCTGTCAATGAACACTCC 3'
T459_00699	F′:5' AGGTTGCCGTTCATGTCCAATG 3'<br>R′:5' CAGCATGGAGTCCAATACCAATACG 3'
T459_15647	F′:5' CAACACGATGCGAGTAATCAGAGG 3'<br>R′:5' CCATGCAGTCCTCGAAGCAATC 3'
T459_20683	F′:5' GTGGTATGGACCTGACCGTGTC 3'<br>R′:5' GAACCATACTGGCTCCTTGAAGTCC 3'
T459_19808	F′:5' GACACTGCTGGACTCTCGGCTG 3'<br>R′:5' GTTGCCGAGGTAGTCAAGTCCAC 3'
T459_05316	F′:5' CAGGCATTGTTGTTAACCGGATC 3'<br>R′:5' GATCAAGAACGGTAGACTTGCTATGTTC 3'
T459_00227	F′:5' CTCGTACAATGATACAGTAGTGGACATCG 3'<br>R′:5' CAGTGTACTGATTCCAACTTGTGCTAGTC 3'

Table S4. qPCR Primer Design for HSP20 Family Gene

Gene ID	Primer
T459_09696	F′ :5' GGTGGAACTTGTAAGGTAACTGCAC 3'<br>R′:5' CTCATGGGTGACATTGGATCTAAC 3'
T459_29869	F′:5' TTCCTTTCCCCACTGATTCTCAA 3<br>R′5' ATGTTTCCACCAGAGGCTGATC 3'
T459_23181	F′:5' CACGTCGCCACGTCTCATTATC 3<br>R′:5' TGAAACCACCTCTAAATATGCCAAC 3'
T459_32220	F′:5' AGAAAGAGGAAGTGAAAGTCGAAGT 3'<br>R′:5' TCTTCTTTAGGAACAGTTACAGTGAGC 3'
T459_13892	F′:5' AATAGAGTGTTGAGAGTGAGCGGAG 3'<br>R′:5' AGTAAAAGAAATAGTGAGCACCCCA 3'
T459_01627	F′:5' AATTTACCTTCAGGACTTGGCAG 3'<br>R′:5' TTCCTTGTGCTCGCCCTTTA 3'
T459_07099	F′:5' ATTAGTGGGAAATGGAAAGCAGAAG 3'<br>R′:5' TTCTTCACCTCTTCTTTAGGGACAGT 3'
T459_00593	F′:5' TTCCGATCCTTCAATACTAACACTC 3'<br>R′:5' ATCTGGTCCATCATGTTCAGCAG 3'
T459_00592	F′:5' ACGGTTTACCCAGTTTCTTCTCAG 3'<br>R′:5' TATATACAGAGCATCAGCATCCTCC 3'
T459_18292	F′:5' GAGGATAGGGTACTTCAGATTAGCG 3'<br>R′:5' GGATTTGACATCAGGCTTCTTCAC 3'
T459_18291	F′:5' CATTCGGTGCCAAGTATCATTC 3'<br>R′:5' GGCTTCCCAGGTTCCAGTTC 3'
T459_17828	F′:5' TCCAAATCAGCATTTCCAGGC 3'<br>R′:5' ATTGAGGTGGAAGAAAACAGGGT 3'
T459_23727	F′:5' GCATCAGCATCCTCCCTCAC 3'<br>R′:5' CTCCGGTCGTTCCGTGTCT 3'
T459_32217	F′:5' GTGAAAGTCGAAGTTGAAGAAGGAA 3'

	R′:5'	CCTCTTCTTTAGGAACAGTTACAGTGAG 3'
T459_32218	F′:5'	AAGGATACTACAGATAAGCGGTGAGA 3'
	R′:5'	TCTTTCGGAACAGTTACAGTGAGC 3'

Figure S1.PCA analysis of All samples

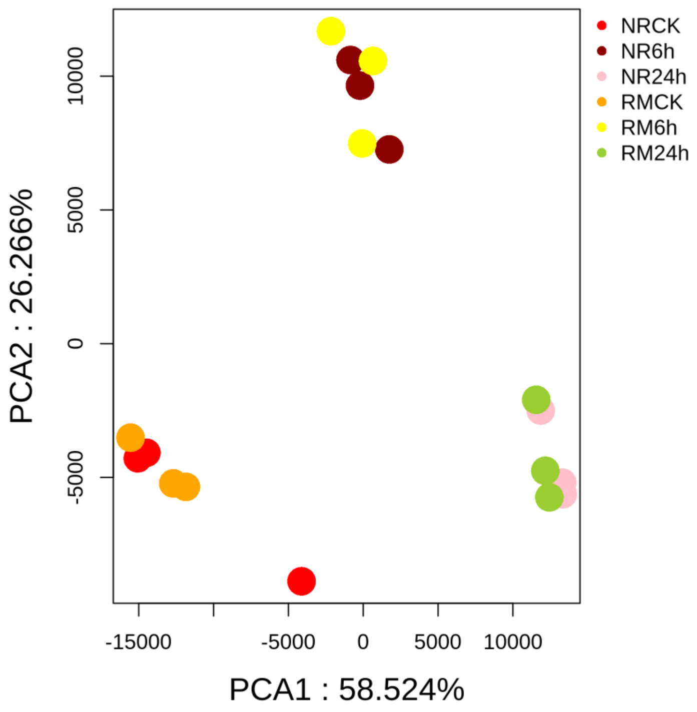

Figure S2.Heatmap analysis of gene expression across various samples

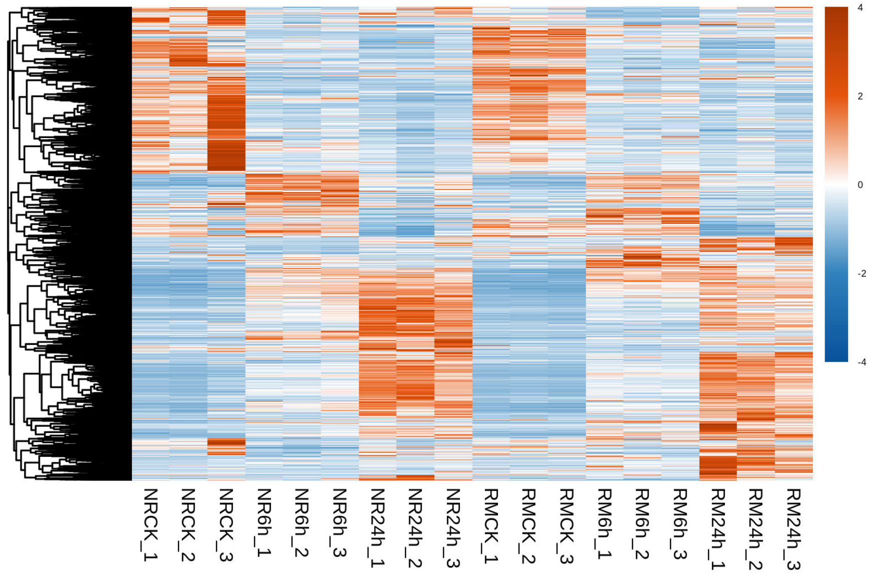

Table S5. **Abbreviations**

The following abbreviations are used in this manuscript:

**ABA**	**Abscisic acid**
ABF	ABA-responsive element binding factor
AP2/ERF	APETALA2/ethylene response factor
APX	Ascorbate peroxidase
ATF6	Activating transcription factor 6
BiP	Binding immunoglobulin protein / GRP78
CAT	Catalase
CaR	Capsicum annuum heat-resistant line
CaS	*Capsicum annuum* heat-sensitive line
CTR1	Constitutive triple response 1
DEGs	Differentially expressed genes
EBF1/2	EIN3-binding F-box protein 1/2
EIN2	Ethylene insensitive 2
EIN3	Ethylene insensitive 3
ER	Endoplasmic reticulum
ERAD	ER-associated degradation
ERE	Ethylene-responsive element (GCC-box)
ERF	Ethylene response factor
ETR	Ethylene receptor
FC	Fold change
FPKM	Fragments per kilobase of transcript per million mapped reads
GST	Glutathione S-transferase
HSF	Heat shock transcription factor
HSE	Heat shock element
HSP / HSP20	Heat shock protein / HSP20 family

HSR	Heat shock response
IRE1	Inositol-requiring enzyme 1
JA	Jasmonic acid
MAPK / MAPKK	Mitogen-activated protein kinase / MAPK kinase
MDA	Malondialdehyde
MPK6	MAP kinase 6
NAC	NAM, ATAF1/2, CUC2 transcription factor family
NCBI	National Center for Biotechnology Information
NPR1	Non-expressor of pathogenesis-related genes 1
PDI	Protein disulfide isomerase
PERK	PKR-like ER kinase
POD	Peroxidase
PYR/PYL	Pyrabactin resistance / PYR-like
qRT-PCR	Quantitative reverse-transcription PCR
RNA-Seq	RNA sequencing
ROS	Reactive oxygen species
SA	Salicylic acid
SAUR	Small auxin-up RNA
SEC61	Sec61 translocon complex
SD	Standard deviation
sHSPs	Small heat shock proteins
SIMKK	SIMK kinase (MAPKK)
SOD	Superoxide dismutase
SnRK2	Sucrose non-fermenting 1-related protein kinase 2
SRP	Signal recognition particle
TGA	TGA transcription factor (bZIP family)

UPR	Unfolded protein response
WRKY	WRKY transcription factor family
